# Dr. Truman Hoffman Squire

**Published:** 1890-02

**Authors:** 


Dr. Truman Hoffman Squire, of Elmira, N. Y., died at his home on Wednesday, November 27, 1889, aged sixty-six years. Dr. Squire has been a prominent figure in professional circles for many years, and his fame has gone beyond the confines of state, and even of nation. He was best known to the medical world as a surgeon, though in his immediate locality his reputation as a physician was not excelled by any of his compeers. Dr. Squire rendered conspicuous service in the late war, and was specially complimented therefor by the Surgeon-General of the Army. He was a writer of great clearness, and his contributions to medical literature have been quoted in two hemispheres. He was a member of several medical societies, local, state, and national, that have adopted suitable tributes to his memory. Not often is the medical profession in the State of New York called to mourn the loss of so great a man.

